# Ten-year survival with analysis of gender difference, risk factors, and causes of death during 13 years of public antiretroviral therapy in rural Kenya

**DOI:** 10.1097/MD.0000000000020328

**Published:** 2020-05-22

**Authors:** Luqman Mushila Hodgkinson, Roselyne Asiko Abwalaba, John Arudo, Michele Barry

**Affiliations:** a Center for Innovation in Global Health; b Stanford University School of Medicine, Stanford University, Stanford, USA; c Masinde Muliro University of Science and Technology School of Medicine; d Department of Clinical Nursing and Health Informatics, Masinde Muliro University of Science and Technology; e Kakamega County Referral Hospital, Kakamega, Kenya.

**Keywords:** AIDS, adherence, antiretroviral therapy, causes of death, gender differences, HIV, sub-Saharan Africa, ten-year survival

## Abstract

Evidence for why antiretroviral therapy (ART) outcomes differ by gender in developing countries has been inconclusive. In this first study to assess 10-year survival on ART in Kenya, our objective was to compare gender differences in survival for those who began ART as adults and as children. Kakamega County Referral Hospital (KCRH) is a tertiary rural hospital that has provided public ART to Kenyans since 2004. All patients enrolled in ART at KCRH who died between July 2004 and March 2017 and a sample of living patients were included in a survival analysis that bootstrapped sampled data. Case–cohort regressions identified adjusted hazard ratios. In total, 1360 patients were included in the study. Ten-year survival was 77% (95% confidence band [CB] 73–81%), significantly different for men (65%; 95% CB: 45–74%) and women (83%; 95% CB: 78–86%) who began therapy as adults. Ten-year survival was intermediate with no significant gender difference (76%; 95% CB: 69–81%) for patients who began therapy as children. Hazard of death was increased for men (hazard ratio [HR] 1.56; 95% confidence interval [CI] 1.13–2.17), infants (HR 2.87; 95% CI 1.44–5.74), patients with consistently poor clinic attendance (HR 3.94; 95% CI 3.19-4.86), and divorced patients (HR 2.25; 95% CI 1.19–4.25). Tuberculosis, diarrheal illnesses, human immunodeficiency virus (HIV) wasting syndrome, and malaria were leading causes of death. Survival was significantly lower for men than for women in all time periods, but only for patients who began therapy as adults, indicating against biological etiologies for the gender mortality difference.

## Abstract 2

Supplemental Digital Content is available in the text

## 1 Introduction

Globally, increasing numbers of people with human immunodeficiency virus (HIV) have been receiving antiretroviral therapy (ART), dramatically increasing life expectancies.^[1–3]^ In Kenya, a nation with 5% adult HIV prevalence,^[4]^ public ART programs have been operating since 2004 at the provincial general hospitals.

Though women are more vulnerable to HIV infection,^[5,6]^ men on ART experience increased mortality in sub-Saharan Africa^[7–14]^ and China^[15]^ though not in Asia-Pacific^[16]^ or Australia.^[17]^ The gender mortality difference in Africa is particularly striking,^[7–14]^ even when corrected for pill count^[12]^ and antiretroviral plasma concentrations.^[13]^ Though 56% of people living with HIV in sub-Saharan Africa are women,^[7]^ an estimated 300,000 men in sub-Saharan Africa died of acquired immunodeficiency syndrome (AIDS)-related illnesses in 2018 compared to 270,000 women.^[7]^ Are men more susceptible to treatment failure due to biological gender differences, for example pharmacokinetic and pharmacodynamic properties of antiretroviral medications^[13,15]^ or decreased thymic ability to regenerate cluster of differentiation 4 (CD4) stocks?^[13]^ This hypothesis is reasonable given gender differences in HIV viral load^[18]^ and progression to neurocognitive impairment.^[19]^ However, behavioral issues associated with concepts of masculinity and stigma,^[20–23]^ programmatic focus on women to reduce mother-to-child transmission of HIV,^[23–25]^ and background gender differences in mortality independent of HIV^[9,26]^ undoubtedly contribute to the observed differences and may be fully explanatory.

As public ART in sub-Saharan Africa became widely available only in the past 15 years, studies of long-term survival on ART have been limited. A recent cohort study at the urban Infectious Diseases Institute in Kampala, Uganda found that 10-year mortality was 23%.^[27,28]^ Prior studies found that 5-year survival in urban Senegal was 75%^[29]^ and 3-year survival for adults in Kenya, Uganda, and Tanzania was 88%.^[30]^ Ten-year survival on publicly administered ART in rural sub-Saharan Africa has not yet been studied, though the vast majority of patients in Africa receive ART through public programs in rural settings.

How survival compares between patients initiated on ART as children and as adults has been uncertain. Pediatric patients initiated on ART in Uganda and Zimbabwe had 1-year mortality of 3% compared to 5% for adults, but these estimates were similar when adjusted for CD4 counts.^[31]^ A meta-analysis found 7% 2-year mortality for pediatric patients in sub-Saharan Africa,^[32]^ though there is wide variation in survival estimates for African children beginning ART.^[33–37]^ Infants have increased hazard of death relative to other children.^[38]^

In this study, we sought to assess 10-year survival for patients receiving publicly administered ART in rural Kenya, comparing gender differences in survival between patients beginning therapy as children and as adults. If gender differences in survival among patients beginning therapy as adults are due mainly to unobserved adherence differences, these may not similarly affect patients accustomed to taking antiretroviral medications since childhood and adolescence.

## 2 Methods

### 2.1 Study population

Kakamega County Referral Hospital (KCRH) was the provincial general hospital for the former Western Province of Kenya and is the largest hospital in this rural region of 7 million people. Of 6133 patients enrolled in ART at KCRH between July 2004 and March 2017, 846 died and 3672 were still active on ART at the time of study, while 1615 had transferred out. When registering, patients were required to supply their contact information, physical address, a sketch of a map to reach their home, and contact information for a friend or relative. A large and active group of community health workers confirmed when patients died or transferred with phone calls and home visits.^[39]^

### 2.2 Data collection and management

Data from time of ART enrollment to time of the study were collected for 1360 patients: all 846 patients who died while on ART at KCRH and 514 active patients from a sample of 524 active patients. Seven patients in the sample declined to participate and 3 had incomplete medical records from repeated transfers. All patients who died while on ART at KCRH were included for a case–cohort analysis to improve statistical efficiency for the number of patients sampled.^[40–42]^ Data were not recorded for patients who left KCRH as it was not possible to obtain their consent. Sources of data included original paper medical records and a partial electronic medical records system, and data were verified with both systems in parallel. Data were stored in a secure Research Electronic Data Capture database.^[43]^

### 2.3 Sampling and informed consent

Patients attended clinic 5 days per week. Adults who were not treatment supporters for children attended clinic every 6 months with medication collection via partner or community health worker every 3 months. Pediatric patients <18 years of age and their treatment supporters were primarily scheduled on Thursdays and Fridays and were seen in clinic every 2 months. Seventeen days were randomly chosen for sampling between October 2016 and March 2017 ensuring that all active patients had a defined probability of being included in the sample: 3 Mondays, 2 Tuesdays, 3 Wednesdays, 6 Thursdays, and 3 Fridays. In total, 524 patients were scheduled for clinic on these sampled days. On days of data collection, all patients scheduled for clinic were given the option to consent for their data to be used in the research. The research was described to each patient privately in the presence of two members of the research team during a discussion lasting at least 15 min. For pediatric patients, consent was requested from a treatment supporter who was a legal guardian and assent was requested from the patient as age appropriate. Written informed consent was provided for all patients in the sample for whom we recorded data. Data for all sampled patients for whom we received informed consent were recorded between January 2017 and March 2017. Accounting for different sampling probabilities by days of the week and the more frequent appointments for pediatric patients with their treatment supporters, normalizing by population proportions, gave approximate inverse sampling probability weights (Table 1).

**Table 1:**
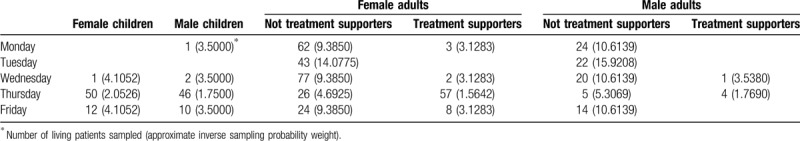
Number of living patients sampled and approximate inverse sampling probability weights.

### 2.4 Model without transfers

The Holmberg predictor^[44]^ generated a pseudo-population of 4517 patients from the 23 strata for living patients (Table 1) and 1 stratum for deceased patients. If the inverse of sampling probability was 7.90, for example, the Holmberg predictor duplicated each data record 8 times with probability 0.90 and 7 times with probability 0.10. This pseudo-population represented all living patients on ART at KCRH at time of consent and all patients who died when on ART at KCRH. Resampling from the Holmberg predictor with a stratified design^[45,46]^ generated 1000 sets of 1360 patients each. Percentile bootstrap confidence intervals from weighted Kaplan–Meier curves were computed from these 1000 sets. Additional Holmberg predictors were generated for patients who began ART as adults and as children by each gender separately, resampling similarly for all sets. Each patient who began ART at KCRH had 1 of 3 outcomes: dying while receiving ART at KCRH, continuing to receive ART at KCRH until the time of data collection, or leaving KCRH. This model underestimated survival as it did not account for the survival prior to leaving of patients who left KCRH.

### 2.5 Model of transfers with constant rate

In this model, the patients who left KCRH were included under the assumption that leaving was stochastic with constant rate. The complete model included 6132 patients: 4517 from the Holmberg predictor and 1615 who left KCRH. To assign censoring times to the patients who left, we considered the sequence of times, *t*_1_, *t*_2_, …, *t*_n_, when deaths or censoring occurred for the patients from the Holmberg predictor measured in days after ART initiation. We assigned N_ti_ = [λ(*t*_*i*_ − *t*_*i*−1_)*R*_*ti*_] patients leaving time *t*_*i*_, where *R*_*ti*_ is the number of patients at risk of dying immediately before *t*_*i*_ including patients who left KCRH. The constant λ was chosen computationally so that the desired number of patients left at each *t*_*i*_ according to the stochastic model and no patients remained after *t*_n_. After finding λ and assigning leaving times, we performed resampling as before with the extra stratum of patients who left KCRH. The model was also applied to patients who began as adults and as children by each gender separately. This model overestimated survival because it assigned longer survival times before leaving than was actually observed.

### 2.6 Model of transfers matching median leaving time

In this model, N_*ti*_ = [λ(*t*_*i*_ − *t*_*i*−1_ + γ/*t*_*i*_)*R*_*ti*_] patients were assigned leaving time *t*_*i*_. This model included a constant γ that allowed the number of patients who left KCRH to decrease with time. The constants γ and λ were chosen computationally so that the median time before leaving was approximately 3 months, the actual median time of leaving for the patients who left KCRH. The model was also applied to patients who began as adults and as children by each gender separately.

### 2.7 Point estimates and confidence bands

Point estimates were derived from the model of transfers that matched median leaving time. Each 95% confidence band (CB) consisted of the lower limit of the 95% confidence interval (CI) from the model without transfers and the upper limit of the 95% CI from the model with transfers at constant rate. Confidence bands accounted for the uncertainty in the distribution of leaving times.

### 2.8 Case–cohort analysis

Stratified case–cohort regressions^[40]^ were fit to the weighted data for the 1360 patients. All covariates used the complete history from the medical records with the most recent state at each time being used. Case–cohort data were obtained by sampling cases and censored individuals separately.^[47]^ Robust variance estimates^[41]^ were conservatively used for confidence intervals though they overestimate variance for case–cohort designs.^[48]^ None of the models reported in this paper had any significant deviations from proportional hazards assumptions as confirmed by tests and plots of Schoenfeld residuals^[42]^ both globally and for each covariate separately.^[49]^ Analyses were performed with Stata Special Edition 14.1.

### 2.9 Adherence debt

When fitting survival models, proportional hazards were satisfied by defining an adherence debt that increased by 1 unit for each clinic appointment missed by more than 1 month and decreased by 1 unit for each 12 contiguous months of met clinic appointments. Measures of adherence that did not allow hazards to return to baseline after behavior change or that returned to baseline more quickly did not satisfy proportional hazards and could not be used in the models.

### 2.10 Ethical approval

This study was approved by the Institutional Review Board for Human Subjects at Stanford University; the Institutional Ethics Review Committee at Masinde Muliro University of Science and Technology; the National Commission for Science, Technology and Innovation, Republic of Kenya; the Kakamega County Director of Health Services; the County Commissioner for Kakamega County; the Director of Education for Kakamega County; and the Ethics and Research Committee at KCRH.

## 3 Results

### 3.1 Baseline characteristics

Of 3672 patients receiving ART at KCRH during data collection, 2387 (65.0%) were adult females, 1003 (27.3%) were adult males, 156 (4.2%) were pediatric females, and 126 (3.4%) were pediatric males. Between 2004 and 2017, 846 patients died while on ART: 422 (49.9%) adult females, 328 (38.8%) adult males, 48 (5.7%) pediatric females, and 48 (5.7%) pediatric males. Of 1615 patients enrolled in ART who left KCRH, 952 patients transferred to other facilities (541 [56.8%] adult females, 321 [33.7%] adult males, 47 [4.9%] pediatric females, and 43 [4.5%] pediatric males) and 663 patients could not be located despite best efforts (349 [52.6%] adult females, 251 [37.9%] adult males, 34 [5.1%] pediatric females, and 29 [4.4%] pediatric males).

Temporal and clinical characteristics of the 1360 sampled patients when enrolling in ART and the regimens initially received are displayed in Table 2. Table S1 in Supplemental Digital Content, displays sociodemographic characteristics including distance of home, parental status of treatment supporters, marital status, and number of children. At enrollment, median age for living women was similar to median age for deceased women (36, interquartile range [IQR] 30-42 vs 35 IQR 30-42). Median ages for men at enrollment were slightly larger, with living men having identical median age as deceased men (40, IQR 32–51 vs 40, IQR 34–48). CD4 counts for deceased men at enrollment were similar to those for deceased women (140 cells/μL vs 157 cells/μL, *t* = 1.17, *P* = .242). Similarly, for adults who were alive at the time of data collection, men had similar CD4 counts as women when beginning ART (243 cells/μL vs 255 cells/μL, *t* = 0.42, *P* = .673). There was no significant gender difference in year of beginning ART for deceased adult patients, with year of beginning ART for deceased men similar to that for deceased women (2010 vs 2009, *t* = 1.44, *P* = .150). However, living men began ART more recently than living women (2013 vs 2011, *t* = 2.88, *P* = .004).

**Table 2:**
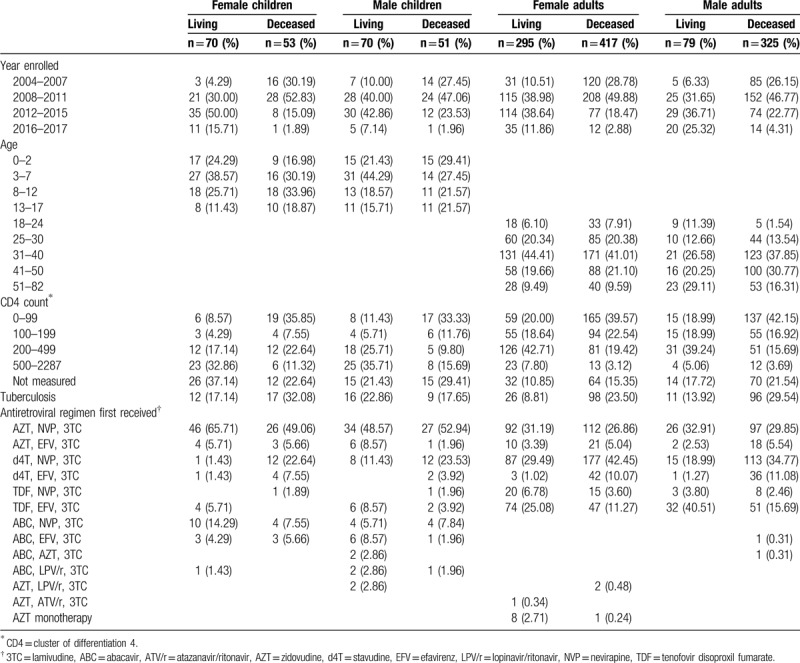
Characteristics of the 1360 patients when enrolling in antiretroviral therapy and the regimens received.

### 3.2 Medications

Only 4.2% of deceased children (4 of 96) and 1.3% of deceased adults (10 of 750) were receiving protease inhibitors at time of death, either lopinavir/ritonavir or atazanavir/ritonavir. All others were receiving first-line regimens consisting of nucleoside/nucleotide reverse transcriptase inhibitors and non-nucleoside reverse transcriptase inhibitors. Of 514 living patients, only 4.9% of children (6 of 122) and 1.8% of adults (7 of 392) were receiving protease inhibitors at time of consent with all others receiving first-line regimens. Nine women received zidovudine as monotherapy during pregnancy in early years of the program. Stavudine was phased out beginning in 2012 with the last patient in the sample switched from stavudine in early 2014.

### 3.3 Causes of death

Tuberculosis was the leading cause of death in adults causing 116 of 750 deaths (15.5%). Among children, diarrheal illness was the leading cause of death (15 of 96 deaths; 15.6%) and tuberculosis was a close second (13 of 96 deaths; 13.5%). Tuberculosis, diarrheal illness, and HIV wasting syndrome were the 3 leading causes of death for both adults and children (Table 3). Malaria, evidenced by positive blood tests, was the fourth leading cause of death (47 of 846 deaths; 5.6%). Other common causes of death were pneumonia, encephalitis, meningitis, Kaposi sarcoma, and urinary tract infections.

**Table 3:**
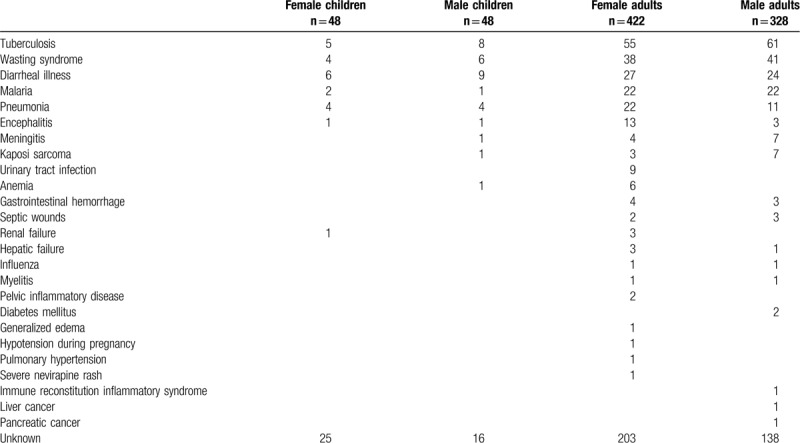
Causes of death.

#### 3.3.1 Survival estimates

**Figure 1:**
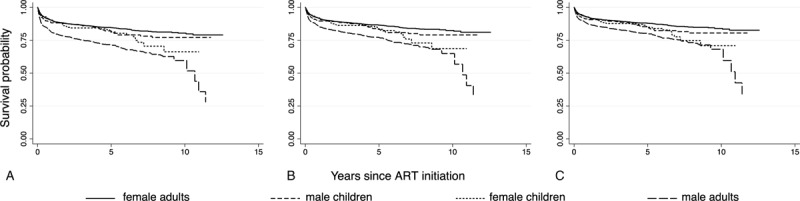
Kaplan–Meier survival curves for patients who enrolled in ART as adults and as children separately by gender. (A) Model without transfers. (B) Model with transfers matching median leaving time. (C) Model with transfers having constant rate of transfer.

Kaplan–Meier survival curves demonstrate high mortality in the first year followed by a slow but steady decline in survival during the following decade (Fig. 1). Overall 10-year survival was 77.3% with 95% CB 72.7–80.6% (Table 4). There was a significant difference in 10-year survival between women (82.5%; 95% CB 78.3–85.8%) and men (65.0%; 95% CB 45.4–73.8%) who began ART as adults, with survival also significantly different at 1 year, 3 years, and 5 years (Table 4). Ten-year survival for patients who began ART as children was intermediate (75.6%; 95% CB 68.6–80.6%) and showed no gender difference.

**Table 4:**
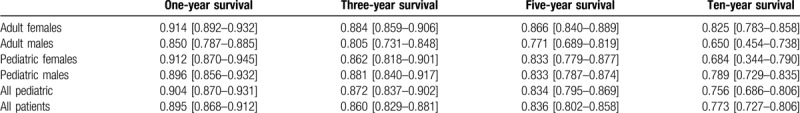
Survival estimates with 95% confidence bands.

#### 3.3.2 Adherence debt

Of deceased patients, 303 (35.8%) had adherence debt at some time in their treatment histories. For these patients, average time with adherence debt was 544 days, an average of 51.6% of treatment time with standard deviation (SD) 27.9%. At time of death, 255 deceased patients (30.1%) had adherence debt. Of sampled living patients, 238 (46.3%) had adherence debt at some time in their treatment histories. For these patients, average time with adherence debt was 704 days, an average of 34.1% of treatment time with SD 22.2%.

#### 3.3.3 Case–cohort analysis

Male patients had significantly higher hazards for death (HR 1.88; 95% CI 1.46–2.41) even when adjusted for age and adherence (Table 5). Patients with adherence debt also had significantly higher hazards for death (HR 3.91; 95% CI 3.17–4.81). Age was not significant when adjusted for gender and adherence.

**Table 5:**
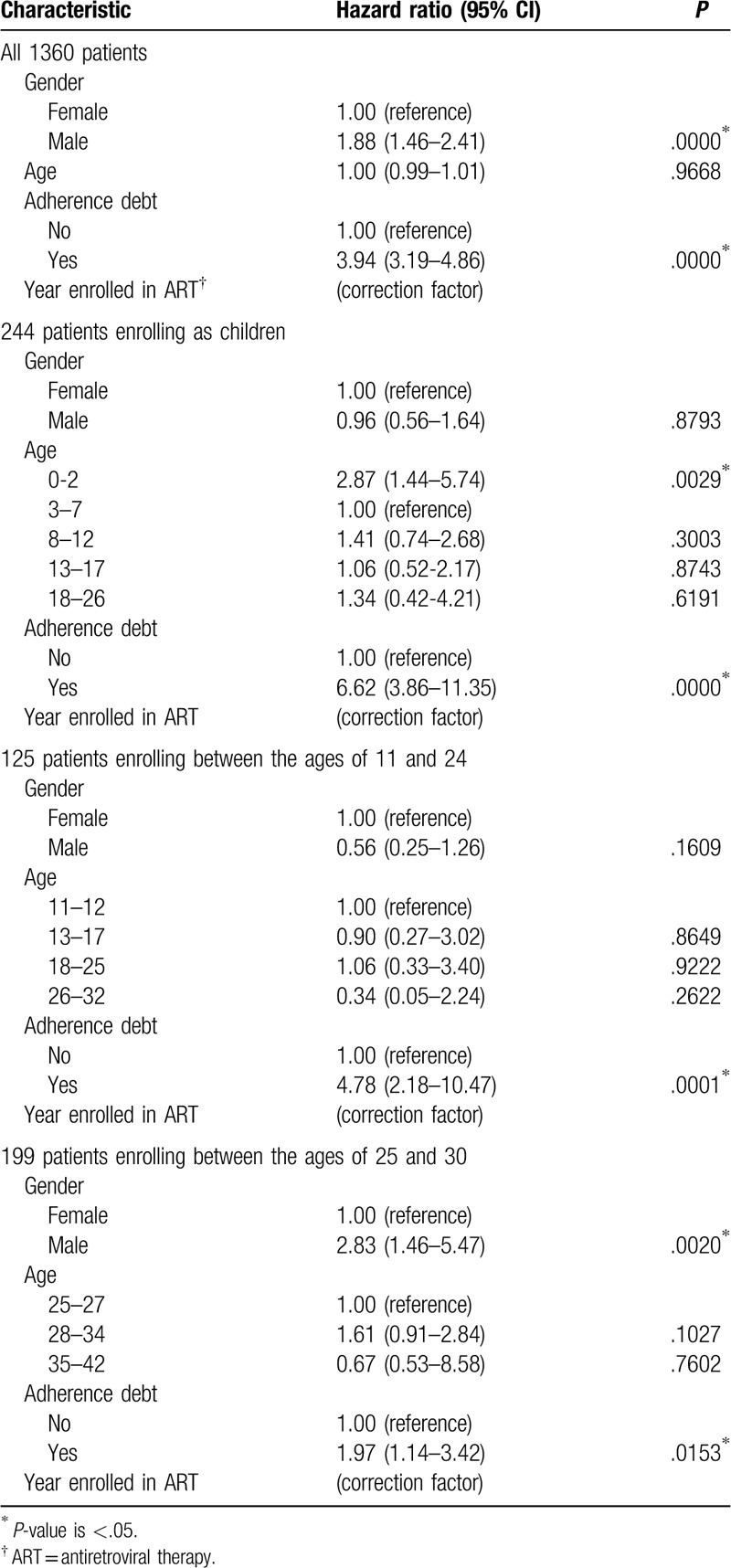
Hazard ratios from case–cohort analysis.

For patients who began ART as children, there was no significant gender difference when adjusted for age and adherence (Table 5). Adherence debt was associated with significant increase in hazard for these young patients (HR 6.62; 95% CI 3.86–11.35). Hazards were also increased for infants less than 3 years of age (HR 2.87; 95% CI 1.44–5.74). For patients who began ART between the ages of 11 and 24, adherence debt was associated with significant increase in hazard (HR 4.78; 95% CI 2.18–10.47), but there was no significant gender difference (Table 5). However, for patients beginning ART between the ages of 25 and 30, male gender was associated with increased hazard of death (HR 2.83; 95% CI 1.46–5.47) independent of adherence debt (HR 1.97; 95% CI 1.14–3.42) (Table 5).

For patients who began ART as adults, hazard decreased significantly as most recent CD4 count increased (HR 0.70; 95% CI 0.63–0.77 for each 100 cells/μL increase), adjusting for gender, age, adherence, and diagnosis of tuberculosis (see Table S2 in Supplemental Digital Content). Hazards were significantly higher for elderly patients at least 74 years of age (HR 6.48; 95% CI 2.99–14.07) and younger adults aged 18 to 38 years (HR 1.53; 95% CI 1.15–2.04), adjusting for CD4 count, gender, adherence, and diagnosis of tuberculosis. Tuberculosis, the leading cause of death among adults, was associated with increased risk of dying at all CD4 counts (HR 2.11; 95% CI 1.50–2.98). Adjusted for CD4 count, increased hazards for male gender (HR 1.56; 95% CI 1.13-2.17) and adherence debt (HR 3.12; 95% CI 2.40-4.05) remained significant.

Hazard ratios increased significantly for viral load at least 2000 copies/mL relative to viral load less than the lowest detectable level (LDL) (see Table S2 in Supplemental Digital Content). Male gender was associated with increased hazard even when adjusted for viral load, age, and adherence (HR 3.96; 95% CI 1.94–8.06).

Patients who were divorced or separated had higher hazards of death (HR 2.25; 95% CI 1.19–4.25) (see Table S3 in Supplemental Digital Content). Number of children did not affect hazard of death except for those with at least nine children (HR 4.33; 95% CI 1.60–11.72). Greater distance of home from KCRH was not associated with increased hazard of death (see Table S3 in Supplemental Digital Content). Hazard of death was not significantly different for patients who received tenofovir disoproxil fumarate relative to zidovudine or stavudine, nor was there any significant difference in hazard between patients who received nevirapine or efavirenz (see Table S4 in Supplemental Digital Content).

## 4 Discussion

Male gender increased risk of death even when adjusted for age, observable adherence, CD4 count, and viral load, but only for patients who began ART as adults. For patients who began ART as children, survival did not differ by gender. Biological gender differences in response to ART have been proposed to explain gender mortality differences,^[13]^ but this is controversial^[15]^ because mortality differences have not been observed in Asia-Pacific^[16]^ or Australia.^[17]^ Measuring antiretroviral plasma concentrations^[13]^ only measures adherence in the previous 2 weeks, and does not account for sporadic adherence outside this window, nor does it account for other behavioral factors such as poor timing, missed doses, or irregular quantities of medications. The gender mortality difference observed in this and prior studies^[8–15]^ was due to patients who began ART in adulthood. Even male patients who began ART as adolescents and young adults, who experienced gender-specific hormones throughout their entire course of treatment, did not have increased mortality relative to their female counterparts after adjusting for observable adherence. In rural Kenya, male gender began to be an independent risk factor for death only for patients beginning ART after the age of 25 years, indicating against explanations due to biological differences in response to therapy that would also affect patients who began ART in adolescence or young adulthood.

Patients who missed clinic by more than 1 month had increased hazard of death, but this hazard did not remain elevated indefinitely. Each month of missed clinic could be forgiven by 12 contiguous months of regular clinic attendance, returning hazards to baseline. This ratio arose naturally from the model of proportional hazards yielding insight into the approximate time required to bring HIV infection under control after lapses in adherence.

Tuberculosis, diarrheal illnesses, and wasting syndrome are leading causes of death for people living with HIV worldwide.^[28,50,51]^ Malaria is recognized as a significant cause of death for pregnant women living with HIV in sub-Saharan Africa.^[52]^ Malaria diagnosed from positive blood smears caused more than 5% of deaths in our study and there was no apparent gender difference. In a smaller sample of 127 adult deaths at the urban Infectious Diseases Institute in Kampala, Uganda,^[27,28]^ cryptococcal meningitis and Kaposi sarcoma were more common causes of death than diarrheal illnesses and malaria, likely due to differences in disease prevalence and treatment options between urban and rural populations.

Integrase strand transfer inhibitors, recommended first-line regimens in the United States for both adults^[53]^ and children at least 2 kilograms in weight,^[54]^ were not available. Protease inhibitors were in short supply and few patients received them. Nevirapine and efavirenz, which most patients received, can be ineffective after a single viral mutation, with cross-resistance between nevirapine and efavirenz.^[55]^ Nevirapine and efavirenz were comparably effective with respect to mortality despite possible differences in viral load suppression,^[56,57]^ and no mortality differences were detected among any available first-line ART regimens. More effective medications with reduced pill burden may lead to improved survival both directly and indirectly through improved adherence.

Strengths of our study include the careful design that included all deceased patients for a comprehensive analysis of causes of death, oversampling of children and young adults to look for any gender differences in this group, and comprehensive recording of CD4 counts, viral loads, antiretroviral medications, missed appointments, and demographic information during 13 years. Limitations of our study include our inability to include all living patients due to budget constraints and transfers, and the restriction to a single site due to legal and budget constraints. Careful bootstrapping and case–cohort design allowed for accurate estimates despite sampling requirements.

In conclusion, our study implies that men are more likely than women to experience mortality when on ART in Kenya, even corrected for observable adherence, but only for those who began ART as adults. For those who began ART as children, there was no detectable gender mortality difference. Our results are valuable in understanding the causes of the gender mortality difference observed in multiple studies in sub-Saharan Africa. Future studies should focus on longer-term follow-up of cohorts who began ART as children and adolescents, looking for gender differences into middle age. The framework of using cohorts who began as children and adolescents to explore causes of the gender mortality difference among adults should be further developed in future studies.

## Acknowledgments

We gratefully acknowledge Dr Ruth Kapanga and her staff at KCRH for ensuring resources for the research; Charles Ayisi, medical records technologist at KCRH, for assisting with medical records and summaries; Prof Charles Chunge for ensuring resources at Masinde Muliro University of Science and Technology School of Medicine and guiding proposals through ethics committees; Josphat Sakwa, Chair of Kakamega County Council of Elders, for community oversight and support; Prof Mike Baiocchi at Stanford University for statistical review and ideas for statistical models; and Prof Eran Bendavid at Stanford University for guidance in manuscript preparation.

## Author contributions

**Conceptualization:** Luqman Mushila Hodgkinson, John Arudo, Michele Barry.

**Data curation:** Luqman Mushila Hodgkinson, Roselyne Asiko Abwalaba, John Arudo.

**Formal analysis:** Luqman Mushila Hodgkinson, Roselyne Asiko Abwalaba, John Arudo, Michele Barry.

**Funding acquisition:** Luqman Mushila Hodgkinson, Michele Barry.

**Investigation:** Luqman Mushila Hodgkinson, Roselyne Asiko Abwalaba, John Arudo, Michele Barry.

**Methodology:** Luqman Mushila Hodgkinson, Roselyne Asiko Abwalaba, John Arudo, Michele Barry.

**Project administration:** Luqman Mushila Hodgkinson, Roselyne Asiko Abwalaba, John Arudo, Michele Barry.

**Resources:** Luqman Mushila Hodgkinson, Roselyne Asiko Abwalaba, John Arudo, Michele Barry.

**Software:** Luqman Mushila Hodgkinson.

**Supervision:** Luqman Mushila Hodgkinson, John Arudo, Michele Barry.

**Validation:** Luqman Mushila Hodgkinson, Roselyne Asiko Abwalaba, John Arudo, Michele Barry.

**Visualization:** Luqman Mushila Hodgkinson, Michele Barry.

**Writing – original draft:** Luqman Mushila Hodgkinson, Roselyne Asiko Abwalaba, John Arudo, Michele Barry.

**Writing – review & editing:** Luqman Mushila Hodgkinson, Roselyne Asiko Abwalaba, John Arudo, Michele Barry.

## Supplementary Material

Supplemental Digital Content
