# Supplementary material for: Ten-year survival with analysis of gender difference, risk factors, and causes of death during 13 years of public antiretroviral therapy in rural Kenya
Source: Medicine (Baltimore). 2020 May 22;99(21):e20328. doi: 10.1097/MD.0000000000020328 (PMC7249944; doi:10.1097/MD.0000000000020328)
Supplement: Supplemental Digital Content [file medi-99-e20328-s001.doc]

**Supplemental Digital Content**

**Table S1: Sociodemographic characteristics of the 1,360 patients when enrolling in ART†**

|  | **Female Children** | | **Male Children** | | **Female Adults** | | **Male Adults** | |
| --- | --- | --- | --- | --- | --- | --- | --- | --- |
|  | **Living** | **Deceased** | **Living** | **Deceased** | **Living** | **Deceased** | **Living** | **Deceased** |
|  | **n=70 (%)** | **n=53 (%)** | **n=70 (%)** | **n=51 (%)** | **n=295 (%)** | **n=417 (%)** | **n=79 (%)** | **n=325 (%)** |
| **Distance of home (km)** | | | | | | | |  |
| 0-4 | 23 (32.86) | 27 (50.94) | 20 (28.57) | 19 (37.25) | 104 (35.25) | 168 (40.29) | 23 (29.11) | 123 (37.85) |
| 5-38 | 44 (62.86) | 25 (47.17) | 48 (68.57) | 30 (58.82) | 182 (61.69) | 233 (55.88) | 50 (63.29) | 188 (57.85) |
| 39-74 | 2 (2.86) | 1 (1.89) | 2 (2.86) |  | 8 (2.71) | 12 (2.88) | 4 (5.06) | 10 (3.08) |
| 75-885 | 1 (1.43) |  |  | 2 (3.92) | 1 (0.34) | 3 (0.72) | 2 (2.53) | 4 (1.23) |
| Not available |  |  |  |  |  | 1 (0.24) |  |  |
| **Treatment supporter** |  |  |  |  |  |  |  |  |
| Parent | 52 (74.29) | 26 (49.06) | 56 (80.00) | 31 (60.78) |  |  |  |  |
| Not parent | 18 (25.71) | 24 (45.28) | 14 (20.00) | 20 (39.22) |  |  |  |  |
| None |  | 3 (5.66) |  |  |  |  |  |  |
| **Marital status** |  |  |  |  |  |  |  |  |
| Never married |  |  |  |  | 31 (10.51) | 49 (11.75) | 10 (12.66) | 22 (6.77) |
| Married |  |  |  |  | 179 (60.68) | 196 (47.00) | 60 (75.95) | 230 (70.77) |
| Divorced/Separated | |  |  |  | 32 (10.85) | 89 (21.34) | 6 (7.59) | 43 (13.23) |
| Widowed |  |  |  |  | 52 (17.63) | 78 (18.71) | 3 (3.80) | 29 (8.92) |
| Not available |  |  |  |  | 1 (0.34) | 5 (1.20) |  | 1 (0.31) |
| **Number of children** |  |  |  |  |  |  |  |  |
| 0 |  |  |  |  | 22 (7.46) | 16 (3.84) | 3 (3.80) | 8 (2.46) |
| 1-5 |  |  |  |  | 206 (69.83) | 121 (29.02) | 48 (60.76) | 73 (22.46) |
| 6-8 |  |  |  |  | 13 (4.41) | 8 (1.92) | 1 (1.27) | 9 (2.77) |
| 9-34 |  |  |  |  |  | 1 (0.24) |  | 4 (1.23) |
| Not available |  |  |  |  | 54 (18.31) | 271 (64.99) | 27 (34.18) | 231 (71.08) |

† ART: antiretroviral therapy

**Table S2: Hazard ratios incorporating CD4**† **count and viral load**

| Characteristic | Hazard ratio (95% CI) | p-value |
| --- | --- | --- |
| **936 patients enrolling as adults who had CD4 measurements** | | |
| CD4 count (each 100 cells/μL) | 0.70 (0.63-0.77) | 0.0000* |
| Gender |  |  |
| Female | 1.00 (reference) |  |
| Male | 1.56 (1.13-2.17) | 0.0076* |
| Age |  |  |
| 18-38 | 1.53 (1.15-2.04) | 0.0031* |
| 39-73 | 1.00 (reference) |  |
| 74-83 | 6.48 (2.99-14.07) | 0.0000* |
| Adherence debt |  |  |
| No | 1.00 (reference) |  |
| Yes | 3.12 (2.40-4.05) | 0.0000* |
| Diagnosis of tuberculosis | 2.11 (1.50-2.98) | 0.0000* |
| Year enrolled in ART‡ | (correction factor) |  |
| **508 patients who had viral load measurements** | | |
| Viral load |  |  |
| Below LDL§ | 1.00 (reference) |  |
| LDL-1,999 | 1.74 (0.40-7.55) | 0.4626 |
| 2,000-49,999 | 4.84 (1.88-12.48) | 0.0011* |
| 50,000-299,999 | 9.16 (2.76-30.48) | 0.0003* |
| 300,000-2,579,064 | 10.11 (2.26-45.20) | 0.0025* |
| Gender |  |  |
| Female | 1.00 (reference) |  |
| Male | 3.96 (1.94-8.06) | 0.0002* |
| Age |  |  |
| 0-17 | 1.04 (0.29-3.74) | 0.9541 |
| 18-38 | 1.00 (reference) |  |
| 39-75 | 1.48 (0.61-3.58) | 0.3860 |
| Adherence debt |  |  |
| No | 1.00 (reference) |  |
| Yes  Year enrolled in ART | 2.16 (1.03-4.57)  (correction factor) | 0.0428* |

* p-value is less than 0.05

† CD4: cluster of differentiation 4

‡ ART: antiretroviral therapy

§ LDL: lowest detectable level

Table S3: Hazard ratios incorporating sociodemographic characteristics

| Characteristic | Hazard ratio (95% CI) | p-value |
| --- | --- | --- |
| **530 patients enrolling as adults who recorded information on marital status and number of children** | | |
| Marital status |  |  |
| Never married | 1.61 (0.70-3.70) | 0.2587 |
| Married | 1.00 (reference) |  |
| Divorced/Separated | 2.25 (1.19-4.25) | 0.0128* |
| Widowed | 0.73 (0.37-1.45) | 0.3682 |
| Number of children |  |  |
| 0 | 0.66 (0.26-1.68) | 0.3807 |
| 1 | 0.76 (0.41-1.40) | 0.3762 |
| 2 | 1.00 (reference) |  |
| 3-8 | 0.82 (0.50-1.37) | 0.4514 |
| 9-34 | 4.33 (1.60-11.72) | 0.0039* |
| Gender |  |  |
| Female | 1.00 (reference) |  |
| Male | 1.98 (1.26-3.11) | 0.0030* |
| Age |  |  |
| 18-38 | 1.51 (1.00-2.27) | 0.0495* |
| 39-73 | 1.00 (reference) |  |
| 74-78 | 6.86 (2.95-15.93) | 0.0000* |
| Adherence debt |  |  |
| No | 1.00 (reference) |  |
| Yes | 2.47 (1.67-3.65) | 0.0000* |
| Diagnosis of tuberculosis | 1.98 (1.25-3.14) | 0.0034* |
| Year enrolled in ART† | (correction factor) |  |
|  |  |  |
| **1,359 patients who recorded home locations** | | |
| Distance from the hospital (per 5 km) | 1.01 (1.00-1.01) | 0.2135 |
| Gender |  |  |
| Female | 1.00 (reference) |  |
| Male | 1.99 (1.53-2.58) | 0.0000* |
| Age |  |  |
| 0-17 | 1.00 (reference) |  |
| 18-38 | 1.41 (1.00-1.99) | 0.0488* |
| 39-73 | 1.09 (0.77-1.53) | 0.6394 |
| 74-83 | 3.86 (1.53-9.73) | 0.0041* |
| Adherence debt |  |  |
| No | 1.00 (reference) |  |
| Yes | 3.96 (3.21-4.90) | 0.0000* |
| Year enrolled in ART | (correction factor) |  |

* p-value is less than 0.05

† ART: antiretroviral therapy

Table S4: Hazard ratios for first-line antiretroviral regimens

| Characteristic | Hazard ratio (95% CI) | p-value |
| --- | --- | --- |
| **903 patients who received standard first-line regimens and never switched regimens, stratified on gender, clinical stage, age, and year enrolled in ART**† | | |
| Nucleoside/nucleotide reverse transcriptase inhibitor | | |
| Zidovudine or Stavudine | 1.00 (reference) |  |
| Tenofovir disoproxil fumarate | 1.17 (0.72-1.91) | 0.5286 |
| Adherence debt |  |  |
| No | 1.00 (reference) |  |
| Yes | 2.09 (1.47-2.98) | 0.0000* |
|  |  |  |
| **903 patients who received standard first-line regimens and never switched regimens, stratified on gender, clinical stage, age, and year enrolled in ART** | | |
| Non-nucleoside reverse transcriptase inhibitor |  |  |
| Nevirapine | 1.00 (reference) |  |
| Efavirenz | 1.22 (0.89-1.68) | 0.2232 |
| Adherence debt |  |  |
| No | 1.00 (reference) |  |
| Yes | 2.12 (1.49-3.01) | 0.0000* |

* p-value is less than 0.05
† ART: antiretroviral therapy
